# Supplementary material for: EnzML: multi-label prediction of enzyme classes using InterPro signatures
Source: BMC Bioinformatics. 2012 Apr 25;13:61. doi: 10.1186/1471-2105-13-61 (PMC3483700; doi:10.1186/1471-2105-13-61)
Supplement: Addtional file 5 — The Java code to format the data files, evaluate and predict. The file enzml_java_code.tar.gz contains the Java code used to format database data to ARFF and XML formats, to execute cross and train-test (jackknife) evaluations and to record evaluation results to database. More information is included in the readme.txt file and the Javadoc files. The code can be used with a MySQL database. To use a different database software, other JDBC drivers might be required. [file 1471-2105-13-61-S5.gz › java_code/utils/doc/cern/jet/random/Pareto.html]

Pareto


---


|  |  |  |
| --- | --- | --- |
| **Overview** \| **Package** \| **Class** \| **Use** \| **Tree** \| **Deprecated** \| **Index** \| **Help** | |  |
| **PREV CLASS**   NEXT CLASS | **FRAMES**    **NO FRAMES**     **All Classes** |  |
| SUMMARY: NESTED \| FIELD \| CONSTR \| METHOD | DETAIL: FIELD \| CONSTR \| METHOD |  |


---


## cern.jet.random Class Pareto

```
java.lang.Object
  cern.colt.PersistentObject
      cern.jet.random.AbstractDistribution
          cern.jet.random.AbstractContinousDistribution
              cern.jet.random.Pareto
```

**All Implemented Interfaces:**: DoubleFunction, IntFunction, java.io.Serializable, java.lang.Cloneable

**Direct Known Subclasses:**: TruncatedPareto

---

``` public class Pareto extends AbstractContinousDistribution ```

Pareto Distribution. See the  math
definition 
animated definition.

p(x) = shape\*scale^shape\*x^{-(1+shape)} for x >= scale,
shape > 0.

The mean is infinite if shape <= 1 and variance is infinite if
shape <= 2.

Instance methods operate on a user supplied uniform random number generator;
they are unsynchronized.

Static methods operate on a default uniform random number generator; they are synchronized. **Version:** : 0.1, 26-Nov-99 **Author:** : Andy Ogielski ato@renesys.com **See Also:**: Serialized Form --- **Constructor Summary** `Pareto(double k, double alpha, RandomElement randomGenerator)`           Constructs a Pareto distribution. | **Method Summary** | | | --- | --- | | `double` | `cdf(double x)`           Returns the cumulative distribution function. | | `double` | `nextDouble()`           Returns a random number from the distribution. | | `double` | `nextDouble(double k, double alpha)`           Returns a random number from the distribution; bypasses the internal state. | | `double` | `pdf(double x)`           Returns the probability distribution function. | | `void` | `setState(double k, double alpha)`           Sets the parameters. | | `static double` | `staticNextDouble(double k, double alpha)`           Returns a random number from the distribution with the given scale = k, and shape = alpha. | | `java.lang.String` | `toString()`           Returns a String representation of the receiver. | **Methods inherited from class cern.jet.random.AbstractDistribution** `apply, apply, clone, makeDefaultGenerator, nextInt` **Methods inherited from class java.lang.Object** `equals, getClass, hashCode, notify, notifyAll, wait, wait, wait` **Constructor Detail** ### Pareto ``` public Pareto(double k, double alpha, RandomElement randomGenerator) ``` : Constructs a Pareto distribution. **Method Detail** ### cdf ``` public double cdf(double x) ``` : Returns the cumulative distribution function. --- ### nextDouble ``` public double nextDouble() ``` : Returns a random number from the distribution. : **Specified by:**: `nextDouble` in class `AbstractDistribution` --- ### nextDouble ``` public double nextDouble(double k, double alpha) ``` : Returns a random number from the distribution; bypasses the internal state. --- ### pdf ``` public double pdf(double x) ``` : Returns the probability distribution function. --- ### setState ``` public void setState(double k, double alpha) ``` : Sets the parameters. --- ### toString ``` public java.lang.String toString() ``` : Returns a String representation of the receiver. : **Overrides:**: `toString` in class `java.lang.Object` --- ### staticNextDouble ``` public static double staticNextDouble(double k, double alpha) ``` : Returns a random number from the distribution with the given scale = k, and shape = alpha. --- | | | | | --- | --- | --- | | **Overview** \| **Package** \| **Class** \| **Use** \| **Tree** \| **Deprecated** \| **Index** \| **Help** | | | | **PREV CLASS**   NEXT CLASS | **FRAMES**    **NO FRAMES**     **All Classes** | | | SUMMARY: NESTED \| FIELD \| CONSTR \| METHOD | DETAIL: FIELD \| CONSTR \| METHOD | | ---
